# Severe Malaria Due to *Plasmodium falciparum* in an Immunocompetent Young Adult: Rapid Progression to Multiorgan Failure

**DOI:** 10.3390/life15081201

**Published:** 2025-07-28

**Authors:** Valeria Sanclemente-Cardoza, Harold Andrés Payán-Salcedo, Jose Luis Estela-Zape

**Affiliations:** 1 Faculty of Health, School of Public Health, Universidad del Valle, Cali 760043, Colombia; valeriasanclemente0@gmail.com; 2 Faculty of Health, Universidad Santiago de Cali, Cali 760035, Colombia; haroldpayan00@usc.edu.co; 3 Health and Movement Research Group, Universidad Santiago de Cali, Cali 760035, Colombia

**Keywords:** malaria, immunity, sepsis, multiple organ failure, *Plasmodium*

## Abstract

*Plasmodium falciparum* malaria remains a major cause of morbidity and mortality, particularly in endemic regions. We report the case of a 21-year-old male with recent travel to an endemic area (Guapi, Colombia), who presented with febrile symptoms, severe respiratory distress, and oxygen saturation below 75%, necessitating orotracheal intubation. During the procedure, he developed pulseless electrical activity cardiac arrest, achieving return of spontaneous circulation after advanced resuscitation. Diagnosis was confirmed by thick blood smear, demonstrating *P. falciparum* infection. The patient progressed to multiorgan failure, including acute respiratory distress syndrome with capillary leak pulmonary edema, refractory distributive shock, acute kidney injury with severe hyperkalemia, and consumptive thrombocytopenia. Management included invasive mechanical ventilation, vasopressor support, sedation-analgesia, neuromuscular blockade, methylene blue, unsuccessful hemodialysis due to hemorrhagic complications, and platelet transfusions. Despite these interventions, the patient experienced a second cardiac arrest and died. This case highlights the severity and rapid progression of severe malaria with multisystem involvement, underscoring the critical importance of early diagnosis and intensive multidisciplinary management. It also emphasizes the need for preventive strategies for travelers to endemic areas and the development of clinical protocols to improve outcomes in complicated malaria.

## 1. Introduction

Malaria is an infectious disease caused by protozoa of the genus *Plasmodium*, transmitted to humans through the bite of infected female Anopheles mosquitoes [1]. It is globally distributed, with the highest incidence in tropical and subtropical regions where environmental conditions favor vector proliferation. In 2022, the World Health Organization estimated approximately 249 million global cases [2].

Among the *Plasmodium* species infecting humans, *Plasmodium falciparum* is associated with the most severe clinical forms. Its pathogenicity is linked to its ability to invade erythrocytes at all maturation stages, reach high parasitemia levels, and promote endothelial cytoadherence, leading to microvascular obstruction. These mechanisms contribute to serious complications such as acute kidney injury, pulmonary edema, severe anemia, and multiorgan failure [3,4].

At the cellular level, *P. falciparum* infection induces structural and functional erythrocyte alterations. Parasitic DNA acts as a potent proinflammatory signal; during hemozoin formation, it is internalized by immune cells and activates Toll-like receptor 9 (TLR9), triggering the release of proinflammatory cytokines and the upregulation of cyclooxygenase-2 (COX-2), which mediates prostaglandin synthesis and the febrile response [5,6]. Cytokines and membrane-derived products released during erythrocyte lysis contribute to systemic manifestations such as headache, myalgia, arthralgia, diarrhea, neurological disturbances, thrombocytopenia, coagulopathies, and immune dysregulation [7].

Although fulminant progression of severe malaria is more common in immunocompromised individuals or those without prior exposure, it may also occur in immunocompetent patients. This report describes the case of a previously healthy young adult who developed *P. falciparum* malaria with rapid clinical deterioration and fatal multiorgan failure.

## 2. Case Report

A 21-year-old male patient with no known history of chronic disease, immunosuppressive therapy, HIV infection, or other conditions associated with immunosuppression presented to the emergency department following recent travel to the Colombian Pacific coast (Guapi). He reported a 5-day history of non-quantified fever, vertigo, and diaphoresis.

Although the thick blood smear confirmed *P. falciparum* infection, parasitemia quantification (% parasitized erythrocytes) was not reported, and molecular confirmation via polymerase chain reaction (PCR) was not performed, limiting the detection of possible mixed Plasmodium infections. Additionally, no further diagnostic tests were conducted to exclude other potential pathogens such as bacterial, viral, or other protozoan infections that could have influenced the clinical course.

**Figure 1:**
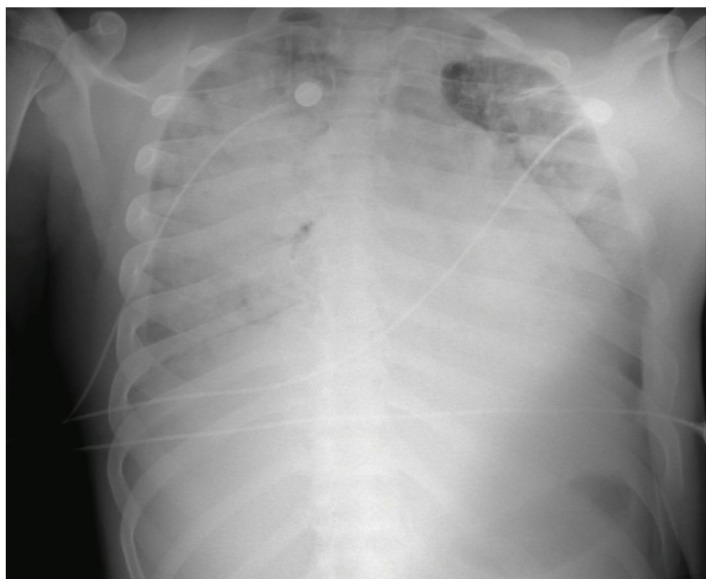
Post-intubation chest X-ray. Anteroposterior chest radiograph showing diffuse bilateral alveolar opacities with loss of lung aeration, consistent with severe acute respiratory distress syndrome (ARDS). Presence of endotracheal tube confirmed in proper position.

Upon admission, the patient exhibited signs of respiratory distress, including the use of accessory muscles and peripheral oxygen saturation (SpO_2_) below 75%, unresponsive to oxygen therapy administered via a non-rebreather mask at 15 L/min. Due to persistent hypoxemia, the patient underwent endotracheal intubation (Figure 1).

During intubation, the patient developed hemodynamic instability progressing to pulseless electrical activity (PEA). Advanced cardiopulmonary resuscitation was initiated, achieving return of spontaneous circulation after 8 min, with restoration of sinus rhythm. Continuous vasopressor support was initiated with norepinephrine (4.0 mcg/kg/min) and vasopressin (20 U/h).

Initial diagnostic work-up included blood tests, chest radiography, thick blood smear, and dengue serology. The thick smear confirmed *P. falciparum* infection, and treatment with artesunate (60 mg) was initiated (Table 1).

In the intensive care unit (ICU), continuous infusions of fentanyl (0.05 mcg/kg/h), midazolam (0.05 mg/kg/h), and propofol (10 mg/mL) were administered for sedation and analgesia. Due to persistent refractory distributive shock, adjunctive therapies with hydrocortisone (100 mg), cisatracurium besylate (10 mg/5 mL), and methylene blue (1%, 10 mg/5 mL) were started.

Arterial blood gas analysis revealed mixed acidosis, severe hypoxemia, hypercapnia, intrapulmonary shunting, and impaired ventilation/perfusion (V/Q) matching. The presence of pink frothy secretions indicated capillary leak pulmonary edema, consistent with a diagnosis of severe acute respiratory distress syndrome (ARDS). Chest radiography demonstrated bilateral infiltrates affecting all four pulmonary quadrants. Prone positioning cycles were indicated based on hemodynamic tolerance (Figure 1).

Renal and metabolic evaluation showed severe hyperkalemia and acute kidney injury, prompting urgent hemodialysis. However, the procedure was unsuccessful due to significant bleeding during Mahurkar catheter insertion, requiring tranexamic acid (500 mg/5 mL) for hemorrhage control. Diuretic therapy with furosemide (20 mg/2 mL) and 20% albumin (50 mL) was initiated. Laboratory results revealed severe consumption thrombocytopenia, prompting transfusion of a platelet pool.

Shortly after transfusion, the patient developed a second episode of cardiac arrest with pulseless electrical activity. A code blue was activated, and advanced cardiopulmonary resuscitation was performed following American Heart Association (AHA) guidelines. Epinephrine (5 vials of 1 mg/1 mL) and calcium gluconate (10%, 10 mL) were administered. Despite 15 min of resuscitation efforts, return of spontaneous circulation was not achieved.

## 3. Discussion

This case report highlights the rapid progression and fatal outcome of *P. falciparum* infection in a previously healthy 21-year-old male, demonstrating the potential severity of this parasitic disease even in immunocompetent individuals outside the traditionally recognized high-risk age group. Despite timely diagnosis, initiation of intravenous artesunate (60 mg every 12 h during the first 24 h, followed by 60 mg daily according to treatment guidelines), and intensive medical support, the patient developed severe systemic complications, including profound hypoxemia, thrombocytopenia, mixed acidemia, distributive shock, and renal and hepatic failure, culminating in multiorgan failure and progressive multisystem failure, leading to death.

Among the five *Plasmodium* species infecting humans, *P. falciparum* is the predominant cause of severe and complicated malaria [8]. Mortality patterns vary with transmission intensity and host age, with the majority of deaths occurring in children under five years of age in sub-Saharan Africa, where this species accounts for approximately 95% of malaria-related mortality [9,10]. In low-transmission settings such as Colombia, clinical severity is influenced by environmental factors including altitude, humidity, and proximity to vector habitats, as well as limited acquired immunity. In such contexts, individuals without prior exposure, including young adults, remain susceptible to severe manifestations [2].

**Figure 2:**
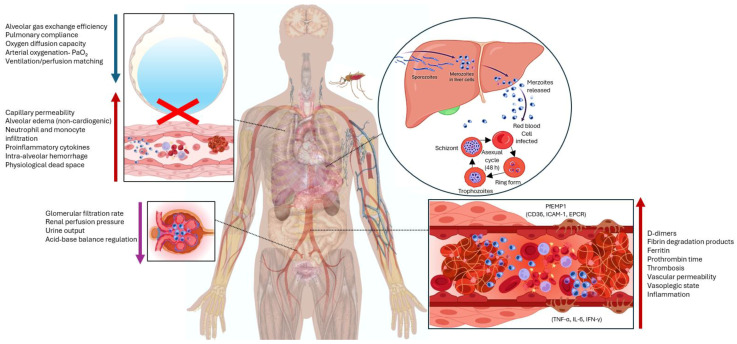
Systemic pathophysiological alterations in severe malaria. Schematic diagram illustrating key mechanisms of severe malaria, including parasite asexual replication in hepatocytes and erythrocytes, PfEMP1-mediated endothelial adhesion and sequestration, systemic inflammation with cytokine release (TNF-α, IL-6, IFN-γ), microvascular thrombosis, and multiorgan dysfunction. Highlighted organ-specific effects include alveolar-capillary leak leading to ARDS, glomerular filtration impairment resulting in acute kidney injury, and hepatic involvement during schizogony. Directional arrows denote pathophysiological changes: red arrows (↑) indicate increased vascular permeability, thrombosis, and systemic inflammation; blue arrows (↓) denote reduced physiological functions, including gas exchange, lung compliance, oxygenation (PaO_2_), renal perfusion, and acid-base balance; purple arrow represent parasite developmental progression and dissemination across organs. The red “X” marks disruption of alveolar gas exchange due to inflammatory alveolar-capillary injury.

Although the precise pathophysiological mechanisms underlying the rapid clinical deterioration in this case cannot be definitively established, several factors may have contributed (Figure 2). The parasite’s high replication rate and the expression of P. falciparum erythrocyte membrane protein 1 (PfEMP1) facilitate cytoadherence to endothelial receptors such as CD36, ICAM-1, and EPCR, promoting microvascular sequestration, impaired tissue perfusion, and endothelial activation [5,11]. PfEMP1 also mediates rosetting and platelet aggregation, further exacerbating microcirculatory obstruction and localized inflammation [12]. These mechanisms may underlie the rapid clinical deterioration observed. Additionally, the outcome may have been influenced by delayed presentation or limited access to advanced supportive therapies such as renal replacement therapy or mechanical ventilation. These contextual factors should be considered when evaluating severe malaria outcomes in non-immune individuals in low-endemic regions.

The host–parasite interaction triggers a dysregulated immune response characterized by a cytokine storm (TNF-α, IFN-γ, IL-6) driven by hemozoin-mediated endothelial activation and mitochondrial metabolic disruption. This results in impaired oxidative phosphorylation, increased anaerobic glycolysis, and metabolic acidosis, contributing to tissue injury [13]. Additional endothelial damage is mediated by CD8+ T-cell cytotoxicity and neutrophil extracellular trap (NET) formation [10].

Systemic inflammation, microvascular sequestration, and prothrombotic activity collectively cause widespread endothelial dysfunction, severe acidosis, and microcirculatory impairment, leading to decreased oxygen delivery and tissue hypoxia. These pathophysiological processes culminate in MODS, affecting vital organs including the kidneys, liver, and lungs. Acute kidney injury (AKI), a frequent complication in severe *falciparum* malaria, affects up to 40% of adults in endemic areas and results from microvascular obstruction by parasitized erythrocytes, immune-mediated glomerular damage, and reduced effective intravascular volume [14,15]. Hepatic injury is also common, manifesting as hyperbilirubinemia, elevated aminotransferases, and jaundice, and correlates with higher incidences of thrombocytopenia, shock, ARDS, and AKI. This injury results from the sequestration of parasitized erythrocytes in hepatic capillaries, causing ischemia and hepatocellular damage [16,17].

In the pulmonary system, the patient developed malaria-associated acute respiratory distress syndrome (MA-ARDS), a highly lethal condition with complex and incompletely understood mechanisms. Inflammation plays a central role by increasing alveolocapillary permeability, which facilitates the extravasation of neutrophils, erythrocytes, and protein-rich plasma into alveolar spaces, resulting in pulmonary edema and severe hypoxemia [18,19]. The combined dysfunction of renal, hepatic, and pulmonary systems led to progressive multiorgan failure and death.

Clinical evidence supports the severity of *P. falciparum* malaria in immunocompetent adults, particularly travelers lacking prophylaxis, who often present with high parasitemia and rapid progression to complications such as cerebral malaria, renal failure, and multiorgan dysfunction. These patients require intensive management with intravenous, artesunate and advanced life support [20,21]. Recent case reports [22,23] describe similar clinical scenarios, emphasizing the critical importance of early diagnosis and multidisciplinary treatment. Organ dysfunction in severe malaria is primarily driven by excessive inflammation and endothelial damage, underscoring the need for integrated therapeutic strategies to improve outcomes.

Several factors may have contributed to the rapid clinical deterioration despite the patient’s previously healthy status. The high parasitic burden and extensive microvascular sequestration likely induced systemic endothelial dysfunction, leading to refractory shock, capillary leak, and multisystem involvement. Laboratory evidence of hyperkalemia, thrombocytopenia, and mixed acidemia corroborates the extent of metabolic and inflammatory disorder typical of severe *P. falciparum* infection.

The patient’s positive dengue IgG serology suggests prior exposure to dengue virus. Although no evidence of acute dengue infection was found, previous dengue infection could hypothetically prime the immune system or exacerbate endothelial dysfunction, potentially influencing the clinical severity of subsequent *P. falciparum* infection. However, this remains speculative given the lack of IgM positivity and confirmatory clinical findings.

Early diagnosis and prompt initiation of antimalarial therapy are essential for improving outcomes in severe malaria. In this case, delayed presentation and lack of prophylaxis likely limited therapeutic efficacy. Despite the administration of intravenous artesunate and supportive measures, progression to multiorgan failure and cardiac arrest ensued, highlighting the narrow window for intervention.

Therapeutic options for severe malaria complicated by multiorgan dysfunction remain limited. Adjunctive treatments such as corticosteroids, vasopressors, neuromuscular blockade, and methylene blue were employed but did not reverse the fatal trajectory. Exchange transfusion, although occasionally considered in cases of hyperparasitemia, was not indicated and remains controversial due to insufficient evidence of benefit.

This case exposes significant gaps in the understanding and management of severe *P. falciparum* malaria in non-immune adults, especially in low-transmission settings. It highlights the necessity for developing adjunctive therapies targeting endothelial dysfunction and inflammatory cascades, as well as predictive tools for the early identification of patients at risk of multiorgan failure. Furthermore, it underscores the importance of reinforcing prophylactic measures, optimizing clinical protocols, and adapting public health responses to evolving epidemiological patterns and emerging high-risk populations.

An important limitation of this report is the absence of Duffy antigen testing. Although Duffy negativity is more strongly associated with *P. vivax* susceptibility patterns, its potential role in modulating the severity of *P. falciparum* infection in specific populations has been suggested. This test was not performed due to limited diagnostic resources in our setting.

## 4. Conclusions

The rapid progression of *P. falciparum* malaria in immunocompetent young adults results from the synergistic interaction of parasite virulence, exaggerated inflammatory response, microvascular dysfunction, and delayed treatment initiation. Timely diagnosis and early intervention are critical to preventing severe outcomes.

To strengthen management, clinical protocols should incorporate the systematic screening of travelers returning from endemic areas, prompt initiation of parenteral artesunate for suspected severe malaria, and standardized multidisciplinary response pathways. Emphasis on pre-travel counseling and strict adherence to chemoprophylaxis guidelines is necessary to reduce imported cases and associated morbidity.

## Figures and Tables

**Table 1:** Laboratory and diagnostic findings in severe malaria with multisystem failure.

Patient’s Values				
Parameter	Reference Range	Emergencies, Date of Entry 6 June 2024	ICU	
			6 June 2024	7 June 2024
Blood Biochemistry				
Hemoglobin (g/dL)	13.5–17.5	16.4	17.2	
Platelets (×10^3^/µL)	150,000–450,000	33,000	42,000	
Leukocytes (×10^3^/µL)	4.5–11.0	8.23	20.37	
Coagulation				
Prothrombin time (seconds)	11.7–15.5		25.7	
Partial thromboplastin time (seconds)	24–45	35.6	40.2	
Electrolytes				
Sodium (mmol/L)	135–145		136	133
Potassium (mmol/L)	3.5–4.5		6.24	6.14
Arterial Blood Gases (mmHg)	Supplemental Oxygen Support	Non-rebreathing Mask	Invasive mechanical ventilation	
FiO_2_		60%	100%	100%
pH	7.35–7.45	7.33	6.96	7.12
pCO_2_ (mmHg)	35–45	41	74	77
PaO_2_ (mmHg)	75–100	58	77	41
HCO^3−^ (mEq/L)	22–26	21.3	16.4	24.8
Base excess (BE)		−17.2	−6.5	
PaO_2_/FiO_2_	>400	96	77	41
Lactic acid (mmol/L)	0.5–2.2	1.5	9.0	2.5
Kidney Function				
Creatinine (mg/dL)	0.7–1.3	1.68	1.10	3.24
Blood urea nitrogen (BUN) (mg/dL)	7–20	23.3	34.3	34.2
Liver Function				
Glutamic-pyruvic transaminase (U/L)	0–45	309	402	
Glutamic-oxalacetic transaminase (U/L)	11–45	795	1190	
Extended Diagnostic Tests				
Thick blood smear for hemoparasites		*Plasmodium falciparum* trophozoites (rings): Positive		
Dengue combo antigen/antibody (NS1Ag/IgM/IgG)		Dengue NS1Ag: Negative Dengue IgM: Negative Dengue IgG: Positive		
Additional Findings				
Thoracic ultrasound (pericardium and pleura)				Bilateral pleural effusion, predominantly on the right side, not susceptible to drainage.

## Data Availability

The authors declare that all data supporting the report are available upon request from the corresponding author.

## References

[1] Olliaro P. (2008). Editorial commentary: Mortality associated with severe *Plasmodium falciparum* malaria increases with age. Clin. Infect. Dis..

[2] Organización Mundial de la Salud (OMS) (2024). Malaria. https://www.who.int/news-room/fact-sheets/detail/malaria.

[3] Instituto Nacional de Salud (INS) (2025). Boletín Epidemiológico Malaria Semana 17-SIVIGILA.

[4] Trampuz A., Jereb M., Muzlovic I., Prabhu R.M. (2003). Clinical review: Severe malaria. Crit. Care.

[5] Smith J.D., Rowe J.A., Higgins M.K., Lavstsen T. (2013). Malaria’s deadly grip: Cytoadhesion of *Plasmodium falciparum*-infected erythrocytes. Cell. Microbiol..

[6] Clark I.A., Cowden W.B. (2003). The pathophysiology of falciparum malaria. Pharmacol. Ther..

[7] Wooldridge G., Nandi D., Chimalizeni Y., O’Brien N. (2020). Cardiovascular Findings in Severe Malaria: A Review. Glob. Heart.

[8] Wassmer S.C., Taylor T.E., Rathod P.K., Mishra S.K., Mohanty S., Arevalo-Herrera M., Duraisingh M.T., Smith J.D. (2015). Investigating the Pathogenesis of Severe Malaria: A Multidisciplinary and Cross-Geographical Approach. Am. J. Trop. Med. Hyg..

[9] Organización Panamericana de la Salud (2025). Malaria.

[10] Moxon C.A., Gibbins M.P., McGuinness D., Milner D.A., Marti M. (2020). New Insights into Malaria Pathogenesis. Annu. Rev. Pathol..

[11] Abdi A.I., Achcar F., Sollelis L., Silva-Filho J.L., Mwikali K., Muthui M., Mwangi S., Kimingi H.W., Orindi B., Kivisi C.A. (2023). *Plasmodium falciparum* adapts its investment into replication versus transmission according to the host environment. eLife.

[12] Yam X.Y., Niang M., Madnani K.G., Preiser P.R. (2017). Three Is a Crowd—New Insights into Rosetting in *Plasmodium falciparum*. Trends Parasitol..

[13] Clark I.A., Alleva L.M., Budd A.C., Cowden W.B. (2008). Understanding the role of inflammatory cytokines in malaria and related diseases. Travel. Med. Infect. Dis..

[14] Koopmans L.C., van Wolfswinkel M.E., Hesselink D.A., Hoorn E.J., Koelewijn R., van Hellemond J.J., van Genderen P.J. (2015). Acute kidney injury in imported *Plasmodium falciparum* malaria. Malar. J..

[15] Mishra S.K., Das B.S. (2008). Malaria and acute kidney injury. Semin. Nephrol..

[16] Jain A., Kaushik R., Kaushik R.M. (2016). Malarial hepatopathy: Clinical profile and association with other malarial complications. Acta Trop..

[17] Fazil A., Vernekar P.V., Geriani D., Pant S., Senthilkumaran S., Anwar N., Prabhu A., Menezes R.G. (2013). Clinical profile and complication of malaria hepatopathy. J. Infect. Public Health.

[18] Hoffmeister B. (2023). Respiratory Distress Complicating Falciparum Malaria Imported to Berlin, Germany: Incidence, Burden, and Risk Factors. Microorganisms.

[19] Sanclemente-Cardoza V., Torres Heredia L.Y., Payan Salcedo H.A., Estela Zape J.L. (2025). Activación De Muerte Celular En Sepsis Y Síndrome De Dificultad Respiratoria Aguda (SDRA). Medicina.

[20] Redditt V., Bogoch I., Rashid M. (2018). A 38-year-old man with fever and a history of malaria. Can. Med. Assoc. J..

[21] Al Farsi F., Chandwani J., Mahdi A.S., Petersen E. (2019). Severe imported malaria in an intensive care unit: A case series. IDCases.

[22] Rodriguez J.A., Roa A.A., Leonso-Bravo A.A., Khatiwada P., Eckardt P., Lemos-Ramirez J. (2020). A Case of *Plasmodium falciparum* Malaria Treated with Artesunate in a 55-Year-Old Woman on Return to Florida from a Visit to Ghana. Am. J. Case Rep..

[23] Teressa M., Purnama A., Henrina J., Wiraatmadja A., Boro A.M.B., Sam C.I.L., Dedang T.A., Cahyadi A. (2023). Severe Malaria in an Adult Patient from Low-Endemic Area in Flores Island, East Nusa Tenggara. Case Rep. Med..

